# A point mutation in the extracellular domain of KIT promotes tumorigenesis of mast cells via ligand-independent auto-dimerization

**DOI:** 10.1038/srep09775

**Published:** 2015-05-12

**Authors:** Yosuke Amagai, Akira Matsuda, Kyungsook Jung, Kumiko Oida, Hyosun Jang, Saori Ishizaka, Hiroshi Matsuda, Akane Tanaka

**Affiliations:** 1 Cooperative Major in Advanced Health Science, Graduate School of Bio-Applications and System Engineering, Tokyo University of Agriculture and Technology, Tokyo, Japan; 2 Laboratory Animal Research Center, The Institute of Medical Science, The University of Tokyo, Tokyo, Japan; 3 Laboratories of Veterinary Molecular Pathology and Therapeutics; 4 Comparative Animal Medicine, Division of Animal Life Science, Institute of Agriculture, Tokyo University of Agriculture and Technology, Tokyo, Japan; 5 Eco-friendly Material Research Center, Korea Research Institute of Bioscience and Biotechnology, Jeonbuk, Korea

## Abstract

Mutations in the juxtamembrane and tyrosine kinase domains of the KIT receptor have been implicated in several cancers and are known to promote tumorigenesis. However, the pathophysiological manifestations of mutations in the extracellular domain remain unknown. In this study, we examined the impact of a mutation in the extracellular domain of KIT on mast cell tumorigenesis. A KIT mutant with an Asn508Ile variation (N508I) in the extracellular domain derived from a canine mast cell tumor was introduced into IC-2 cells. The IC-2^N508I^ cells proliferated in a cytokine-independent manner and showed KIT auto-phosphorylation. Subcutaneous injection of IC-2^N508I^ cells into the dorsal area of immunodeficient BALB/c-*nu/nu* mice resulted in the formation of solid tumors, but tumor progression was abrogated by treatment with a tyrosine kinase inhibitor (STI571). In addition, the N508I mutant KIT protein dimerized in the absence of the natural ligand, stem cell factor. Structure modeling indicates that the increased hydrophobicity of the mutant led to the stabilization of KIT dimers. These results suggest that this extracellular domain mutation confers a ligand-independent tumorigenic phenotype to mast cells by KIT auto-dimerization that is STI571-sensitive. This is the first report demonstrating the tumorigenic potential of a mutation in the extracellular domain of KIT.

KIT is a type-III receptor tyrosine kinase encoded by the c-*kit* gene that plays important roles in the maintenance and proliferation of melanocytes, interstitial cells of Cajal, and hematopoietic cells such as stem cells, hematopoietic progenitors, and mast cells^1^,^2^,^3^. Binding to the stem cell factor (SCF) leads to KIT dimerization, resulting in the phosphorylation of tyrosine residues and activation of downstream signaling molecules^4^,^5^,^6^. Mutations in KIT, especially in the juxtamembrane or tyrosine kinase domains, have been detected in a wide variety of tumors including leukemia, gastrointestinal stromal tumors (GISTs), melanomas, and mast cell malignances^7^,^8^,^9^,^10^. These mutations have been shown to result in KIT autophosphorylation, even in the absence of SCF binding^11^,^12^,^13^. Specifically, insightful studies by Kitamura *et al*.^12^,^13^, in which the roles of mutations in the juxtamembrane and tyrosine kinase domains in the tumorigenesis of mast cells were investigated, revealed a critical role for these mutations in promoting mast cells tumorigenesis, both *in vitro* and *in vivo*. For their analyses, they utilized the interleukin 3 (IL-3)-dependent IC-2 mast cell line^14^, which was derived from bone marrow-derived cultured mast cells^13^. Because of their origin, IC-2 cells possess characteristics similar to normal mast cells and differentiate in response to several stimulators, including IL-4 and granulocyte-macrophage colony-stimulating factor^15^. A distinctive feature of IC-2 cells is that they lack KIT expression, enabling researchers to compare the functions of wild-type and mutant KIT proteins in cells with a mast cell phenotype. Thus, IC-2 cells are useful for investigating the effects of KIT mutations on mast cell tumorigenesis or phenotypic alterations. Kitamura *et al*.^12^ performed crosslinking experiments to explore the activation mechanism of KIT mutants. They found that a mutation in the KIT juxtamembrane domain causes autophosphorylation and homodimerization even in the absence of SCF, while a mutation in the tyrosine kinase domain leads to autophosphorylation irrespective of dimer formation or SCF binding. However, the roles of alternative mutations in supporting mast cell tumorigenesis or proper KIT protein conformations are not fully understood.

Mutations in the KIT extracellular domain have been reported not only in mast cell malignances, but also in various other cancers such as acute myeloid leukemia (AML) and GIST^16^,^17^,^18^,^19^,^20^,^21^. Those studies revealed that mutations in the extracellular domain cause KIT autophosphorylation, although it is unclear whether these mutations directly promote tumorigenesis *in vivo*. Interestingly, most mutations in the KIT extracellular domain exist in the fifth immunoglobulin-like (Ig-like) domain, which is encoded by sequences in exon 8 and 9^16^,^17^,^18^,^19^,^20^,^21^. Most mutations reported in other type III receptor tyrosine kinases, such as platelet-derived growth factor receptor-α or colony-stimulating-factor 1 receptor, have also been identified in the Ig-like domains close to the cell membrane^22^,^23^. Thus, it is possible that KIT mutations in the fifth Ig-like domain may play a key role in the regulation of KIT conformation, thus determining its phosphorylation status. A molecular-level approach will aid in the understanding of this possibility and in the development of novel selective inhibitors.

Compared to humans, dogs are more frequently diagnosed with mast cell malignancies^24^. Our group^25^ and others^26^,^27^recently noted that canine mast cell tumors (MCTs), which account for approximately 20% of all cutaneous tumors in dogs, show mutations in the extracellular domain of KIT. We investigated the direct contribution of the extracellular domain mutation to mast cell tumorigenesis using retrovirally transduced IC-2 mast cells as a model. Our results demonstrated that a point mutation in the extracellular domain of KIT leads to the cytokine-independent proliferation of mast cells, both *in vitro* and *in vivo*. Moreover, we report for the first time that this mutation induces the autophosphorylation of KIT by forming a dimer, due to the enhanced hydrophobicity and activity even in the absence of SCF. These results raise the possibility for a new molecular strategy targeting type-III receptor tyrosine kinases with a mutation in the extracellular domain.

## Results

### Establishment of IC-2 sublines and characterization of their *in vitro* growth properties

**Figure 1:**
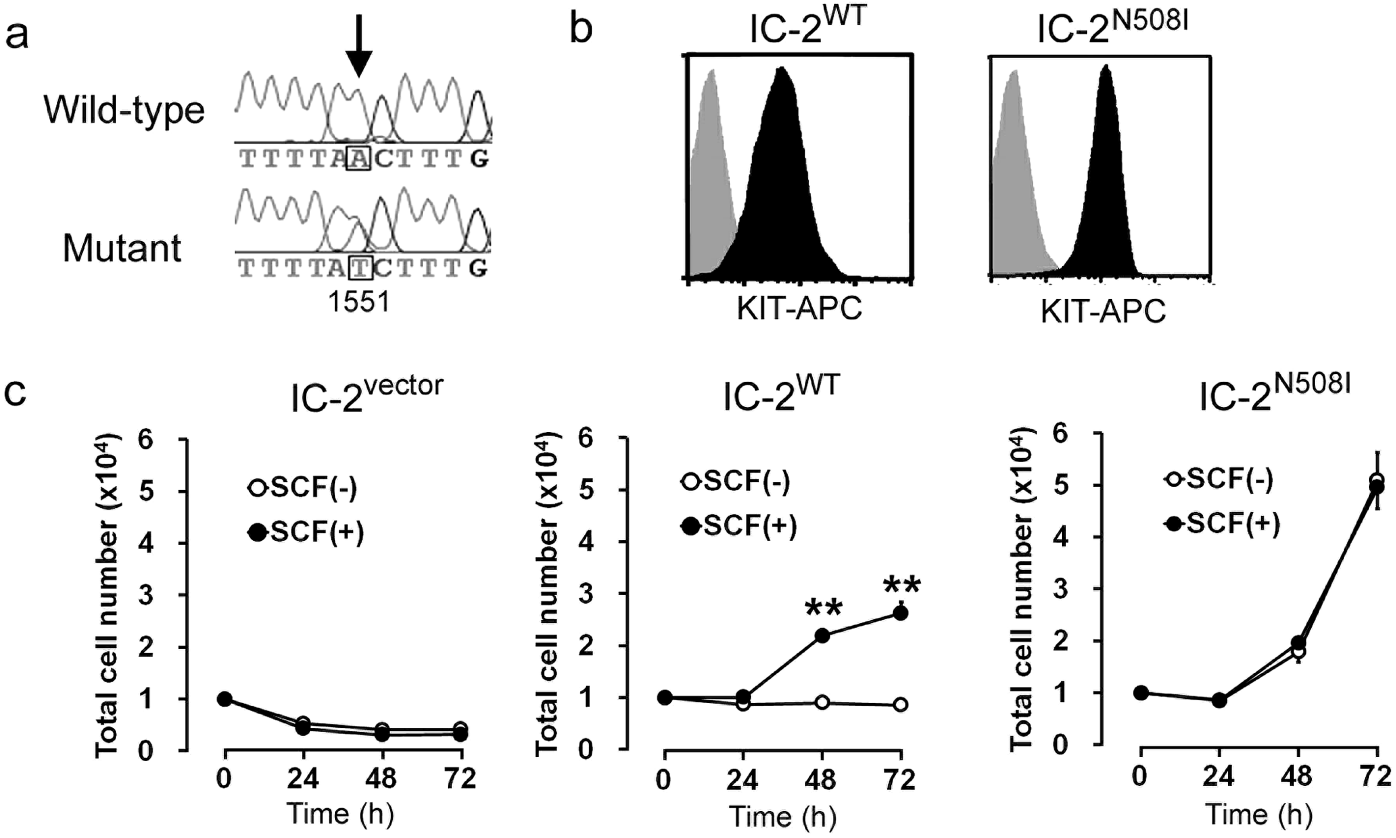
Characterization of IC-2^N508I^ cells. (a) sequence of the c-*kit* gene. The arrow indicates a heterozygous point mutation in codon 508 (1551 A > T) from a clinical sample from a canine diagnosed with MCT. The base number corresponds to GenBank accession no. AF044249. (b) representative flow cytometry analysis data. Cell surface KIT expression in the indicated IC-2 sublines was detected using an anti-KIT-APC antibody (black), and KIT expression in IC-2^vector^ cells was used as a negative control (gray). (**c**) growth curves of IC-2 sublines in the presence or absence of 10 ng/mL SCF. Data represent means ± standard deviations (SD) of 3 independent experiments (n = 5 at each time point). ** *p* > 0.01, compared to SCF (-) cells at each time point.

When analyzing c-*kit* sequences in 13 surgically removed canine MCT samples, an 1551 A>T point mutation, resulting in an Asn508Ile amino acid change (N508I), was discovered in a single specimen from dogs presented to the Animal Medical Center in Tokyo University of Agriculture and Technology (Fig. 1a). Although the N508I mutation has been reported in dog MCTs by several groups^26^,^27^, the contribution of this mutation to mast cell tumorigenesis remains unclear. Cells isolated from the tumor included abundant basophilic granules in their cytosol that showed metachromasia by acid toluidine blue staining (Fig. S1a). Cultured primary cells from the tumor showed both morphological and genetic characteristics of mast cells. For example, expression of dog mast cell protease 3 (dMCP-3^28^, Fig. S1b) was observed, as was phosphorylation of KIT receptors (Fig. S1c). In addition, no other mutations in the c-*kit* gene were identified in the tumor, except for 1551 A>T (Fig. S1d). To determine whether the mutation promotes mast cell tumorigenesis, we established an IC-2 mast cell subline expressing the N508I mutant KIT (IC-2^N508I^ cells; Fig. 1b), using a procedure reported by Hashimoto *et al.*^13^ As controls, wild-type KIT-expressing (IC-2^WT^ cells) and mock-transfected (IC-2^vector^ cells) sublines were also established. Because the parental IC-2 cells are dependent on IL-3 for growth and do not express KIT receptors^14^, IC-2^vector^ cells did not proliferate in the absence of IL-3, regardless of SCF addition (Fig. 1c). Next, the growth of IC-2^WT^ and IC-2^N508I^ cells was examined under IL-3-free conditions. As shown in Fig. 1c, IC-2^N508I^ cells proliferated independently of SCF, while IC-2^WT^ cells only grew in the presence of SCF. The doubling time of IC-2^N508I^ cells in the absence of SCF was approximately 26.1 h (Fig. 1c).

### STI571 sensitivity of IC-2^N508I^ cells

**Figure 2:**
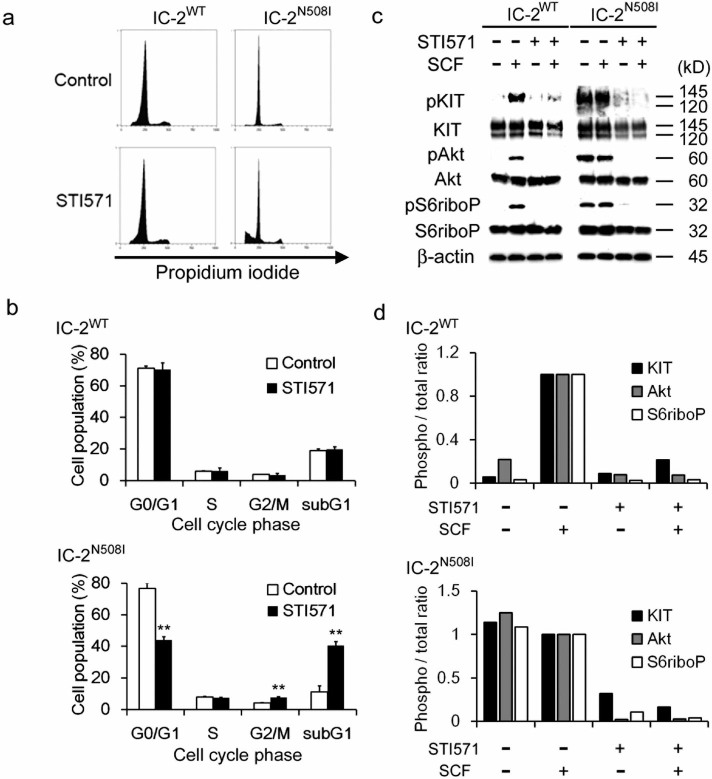
STI571 sensitivity and PI3K signaling activity in IC-2^N508I^ cells. (a, b) representative cell cycle analysis data and distribution of cells in each cell cycle phase as a function of STI571 treatment. Each subline was serum-starved overnight and then cultured with or without 250 nM STI571 for 24 h. After incubation, cells were fixed with 70% ethanol and stained with propidium iodide. Each data point represents the mean ± SD of 3 independent experiments with duplication. ** *p* > 0.01, relative to untreated control cells. (c) western blot analysis of each IC-2 subline and (d) quantification of the ratio of phosphorylated/total amounts of each protein. Cells were serum-starved overnight, cultured for 4 h in the presence or absence of 10 ng/mL SCF and/or 250 nM STI571, and expression of the indicated proteins was analyzed in western blots. The phosphorylated/total ratios of cells cultured in the presence of SCF were set to 1.

STI571 is a well-known competitive inhibitor of adenosine 5′-triphosphate (ATP) that suppresses the activation of several tyrosine kinases, including KIT^29^,^30^. To determine whether the N508I KIT mutation affects the sensitivity of cells to STI571, a water-soluble tetrazolium salt (WST) assay was performed. The assay showed inhibitory concentration (IC_50_) values of 123.7 ± 15.3 nM in IC-2^N508I^ cells after STI571 treatment. However, the corresponding IC_50_ values for IC-2^WT^ cells following STI571 treatment were above the detection limit (> 1 μM). Cell cycle analysis was conducted to confirm the inhibitory effect of STI571 on IC-2^N508I^ cells. The proportion of sub-G1 (apoptotic) IC-2^N508I^ cells was increased upon STI571 treatment; however, these alterations were not observed in IC-2^WT^ cells (Figs 2a and b). Western blots were performed to evaluate these results in terms of differences in effector protein phosphorylation. Because the phosphoinositide 3-kinase (PI3K) signaling pathway is activated downstream of KIT activation^31^,^32^, we assessed the phosphorylation levels of the Akt and S6 ribosomal proteins, which are key signaling molecules in the PI3K pathway. As shown in Figs 2c and d, SCF-dependent activation of these proteins was observed in IC-2^WT^ cells. In contrast, phosphorylation of these proteins occurred in IC-2^N508I^ cells independently of SCF addition (Figs 2c and d). To study the inhibitory effect of STI571 on the phosphorylation of these proteins, cells were treated with STI571 in the presence or absence of SCF. As shown in Figs 2c and d, phosphorylation of each protein examined was strongly inhibited by STI571 in both IC-2^WT^ and IC-2^N508I^ cells.

### Dimerization of KIT in IC-2 sublines

**Figure 3:**
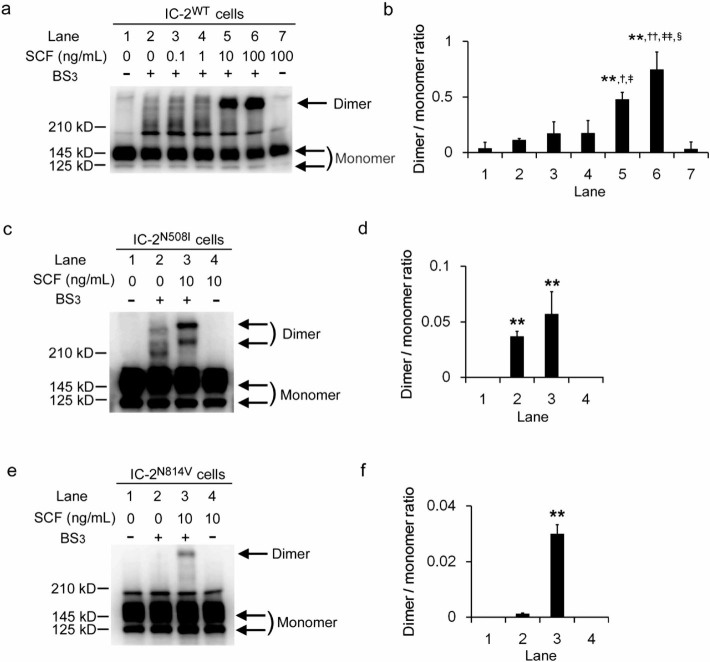
KIT dimerization in IC-2 sublines. (a) western blot analysis of IC-2^WT^ cells for KIT. Cells were treated with indicated concentrations of SCF, followed by the BS_3_ crosslinker (1 mM). (b) the mean dimer/monomer ratios ± SD of KIT observed in 3 independent experiments are indicated. Lane numbers correspond to those shown in Figure 3a. Arrows indicate monomeric or dimeric forms of KIT. **, *p* > 0.01 compared to lane 2; , †, ††, *p* > 0.05, 0.01 compared to lane 3; , ‡, ‡‡, *p* > 0.05, 0.01 compared to lane 4; and §, *p* > 0.01 compared to lane 5, respectively. (c, e) western blot analysis of IC-2^N508I^ cells (**c**) and IC-2^N814V^ cells for KIT (**e**). Cells were treated with the indicated concentrations of SCF and/or 1 mM BS_3_. Arrows indicate monomeric or dimeric KIT. (d, f) the mean dimer/monomer ratios ± SD of KIT from 3 independent experiments are shown. Lane numbers correspond to those shown in Figure 3c and e, respectively. ** *p* > 0.01 compared to lane 1.

Kitayama *et al.*^12^ reported that KIT dimerization, which is necessary for autophosphorylation, occurs in a juxtamembrane domain KIT mutant in the absence of SCF, while a tyrosine kinase domain KIT mutant is activated without dimerization. Dimerization of wild-type KIT was observed in IC-2^WT^ cells in the presence of SCF, in a dose-dependent manner. In IC-2^WT^ cells, the basal level of KIT dimerization without SCF addition was low, and dose-dependent increase of dimer formation was observed by SCF supplementation (Figs 3a and b). Clear dimerization was detected in the presence of 10 ng/mL SCF (Figs 3a and b). To determine whether ligand-independent dimerization exists in N508I KIT, we next treated IC-2^N508I^ cells with bis(sulfosuccinimidyl) suberate (BS_3_) in the presence or absence of 10 ng/mL SCF and subjected to cell lysates to western blot analysis. SCF-independent dimerization was observed in IC-2^N508I^ cells (Figs 3c and d). In contrast, IC-2^N814V^ cells, which express a KIT mutant bearing a point mutation in the tyrosine kinase domain, only dimerized in the presence of SCF as previously reported (Figs 3e and f)^12^. While a single band corresponding to KIT dimers was detected in IC-2^N814V^ cells only in the presence of SCF (Fig. 3e), multiple bands were observed in IC-2^N508I^ cells, both in the presence or absence of SCF (Fig. 3c).

### Tumorigenicity and STI571 sensitivity of IC-2^N508I^ cells

**Figure 4:**
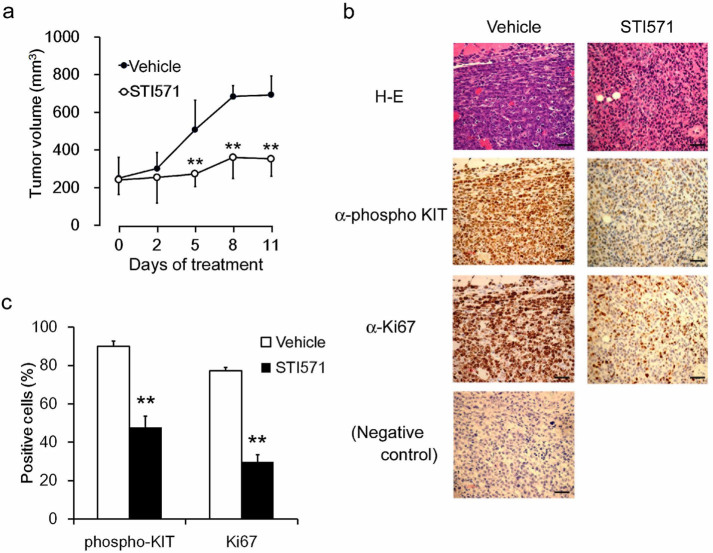
*In vivo* growth and STI571 sensitivity of IC-2^N508I^ cells. (a) growth curves of IC-2^N508I^ cells *in vivo*. A total number of 5 × 10^6^ cells were injected subcutaneously into the flanks of BALB/c-*nu/nu* mice, and tumor sizes were measured every 2 or 3 days. STI571 (100 mg/kg) was administered orally daily, starting 10 days after tumor cell transplantations (Day 0 in the graph). Each data point represents the mean ± SD for 6 animals in each group. ** *p* > 0.01, compared to vehicle-treated mice. (b) immunohistochemical analysis of IC-2^N508I^ cells. Tissues were collected on Day 11, and phospho-KIT and Ki-67 staining was conducted. Original magnification, × 200. Bar; 100 μm. (c) percentages of phospho-KIT- or Ki-67-positive cells are indicated as means ± SD obtained from 5 randomly selected microscopic fields. ** *p* > 0.01, compared to vehicle-treated.

Next we investigated the tumorigenicity of IC-2^N508I^ cells *in vivo* by subcutaneously injecting them into the right and left flanks of immunodeficient BALB/c-*nu/nu* mice. Although IC-2^WT^ cells were not tumorigenic (data not shown), IC-2^N508I^ cells proliferated in mice and formed solid tumors, which were increased in volume at the injection sites (Fig. 4a). KIT phosphorylation and high expression of cell growth marker Ki-67^33^ were detected in tumor tissues by immunohistochemistry (Fig. 4b). The inhibitory effect of STI571 was also examined in these *in vivo* models. Daily oral administration of 100 mg/kg STI571 attenuated the growth of xenograft IC-2^N508I^ tumors by approximately 50% (Figs 4b and c). All mice were sacrificed at 11 days after STI571 administration, after which tumor tissues were collected. At this point, most tumor tissues from STI571-treated mice were necrotic. In these tumor tissues, KIT phosphorylation and Ki-67 positivity were markedly reduced compared to levels observed in vehicle-treated mice (Fig. 4c).

### Structural modeling of wild-type and N508I KIT proteins

**Figure 5:**
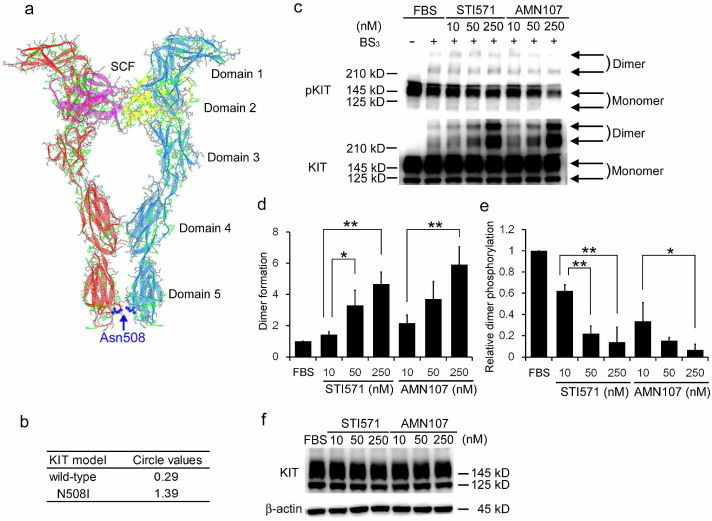
Structure model of the extracellular domain of canine KIT and the effect of KIT inhibitors on dimerization. (a) modeling of the extracellular KIT domain in the SCF-bound condition. Red and blue ribbons indicate main-chains, green wires indicate the hydrophobic residues, and the blue ball & stick models indicates the residue Asn508. Pink and yellow ribbons indicate stem cell factor. (b) comparison of circle values between wild-type and N508I mutant canine KIT. Circle values for residue 508 in wild-type and N508I canine KIT are indicated. (c) western blot analysis of IC-2^N508I^ cells treated with STI571 or AMN107. Cells were treated with either reagent for 4 h, followed by chemical crosslinking with BS_3_. The monomeric and dimeric forms of KIT are indicated with arrows. (d) the relative mean dimer/monomer ratios ± SD from 3 independent experiments are shown. The dimer/monomer ratios of cells cultured in FBS-containing medium were set to 1. *, **, *p* > 0.05, 0.01, compared to treatment with 10 nM STI571 or AMN107, as indicated. (e) relative mean dimer phosphorylation levels ± SD from 3 independent experiments are indicated. The dimer/monomer ratio observed in cells cultured in FBS-containing medium was set to 1. *, **, *p* > 0.05, 0.01 compared to cells treated with 10 nM of the indicated reagents. (f) western blot analysis of IC-2^N508I^ cells treated with STI571 or AMN107. Cells were treated by each agent for 4 h and lysed without BS_3_ treatment.

The data above suggest that dimerization of wild-type KIT led to the activation of the receptor and downstream signaling molecules only in the presence of SCF, while ligand-independent dimerization of N508I KIT resulted in tumorigenesis by causing aberrant signaling activations. To determine the molecular mechanism of N508I KIT dimerization, the structures of both wild-type and N508I KIT were simulated. The dimeric form of wild-type canine KIT was modeled based on the known crystal structure of human KIT^34^. Molecular modeling predicted that Asn508 residues (located in the fifth Ig-like domain) faced each other and formed hydrogen bonds in the dimerized state (Fig. 5a). Circle values, reflecting the stability of modeled structures^35^, were calculated to compare differences in stability following dimerization. Under SCF-free conditions, the circle value for amino acid residue Asn508 was 1.39 for the N508I KIT mutant, which was markedly higher than that for the wild-type KIT (0.29; Fig. 5b). An increase in the circle values for the extracellular domain-mutant KIT was also confirmed in human KIT, which has been reported in AML and GIST patients (Table S1)^16^,^17^,^18^,^19^,^20^.

### Effects of KIT inhibitors on KIT dimerization

Finally, to clarify the effect of KIT inhibitors on KIT dimerization, IC-2^N508I^ cells were treated with STI571, followed by treatment with a crosslinker BS_3_. We observed that the degree of KIT dimerization was markedly increased by STI571 treatment, while KIT phosphorylation was not (Figs 5c, d and e). To exclude the possibility of structure-specific KIT crosslinking by STI571, the effect of another ATP-competitive KIT inhibitor AMN107^36^ was examined. The IC_50_ value of AMN107 in IC-2^N508I^ cells was 10.1 ± 0.4 nM. A dose-dependent increase in KIT dimerization was also observed following AMN107 treatment (Figs 5c, d and e), although the total amount of KIT protein expressed was unaffected (Fig. 5f). In addition, we studied the effects of KIT inhibitors on *de novo* KIT synthesis and internalization by RT-PCR and flow cytometry and observed that neither mRNA nor surface KIT expression levels were altered by STI571 and AMN107 treatment (Figs S2a, b and c).

## Discussion

**Figure 6:**
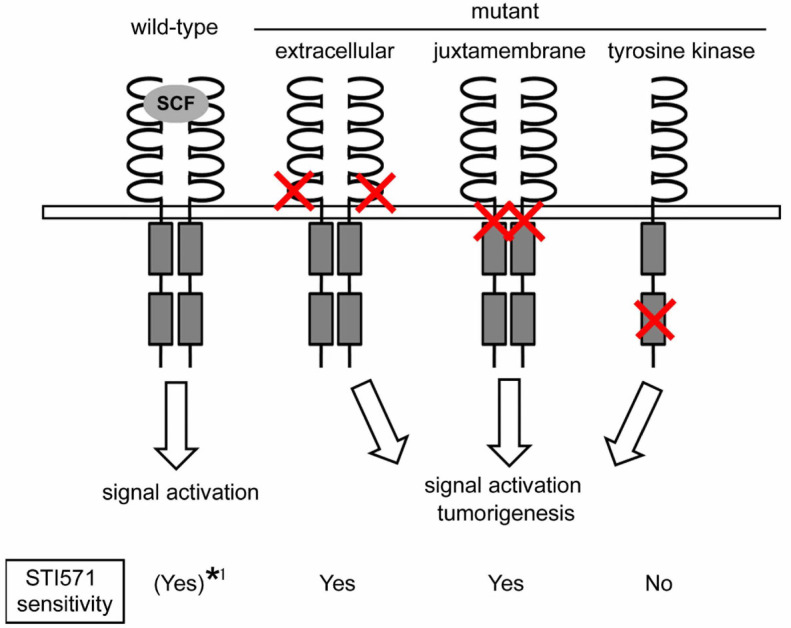
Schematic representation of mutant KIT phenotypes. This diagrammatic representation describes the correlation of known KIT mutations with activation patterns. While wild-type KIT dimerizes and gets activated only in the presence of SCF, KIT with mutations in either the extracellular or the juxtamembrane domain dimerizes and becomes activated independently of SCF binding. The variant KIT with a mutation in the tyrosine kinase domain does not require SCF stimulation or dimerization for activation. STI571 sensitivities are also indicated. *1, sensitive to STI571, but less sensitive than mutant KIT.

In this study, we demonstrated that an Asn508Ile mutation in the extracellular domain of KIT is tumorigenic in mast cells. Including our findings, the schema of KIT mutants with their activation patterns and STI571 sensitivities is presented in Fig. 6. The N508I mutant KIT conferred cytokine-independent growth of IC-2 cells and induced tumorigenicity both *in vitro* and *in vivo*, as a result of SCF-independent KIT dimerization and autophosphorylation.

Replacement of Asn508 residue with the hydrophobic amino acid isoleucine resulted in the enhancement of circle values, indicating that the hydrophobicity of residue Asn508 stabilizes the KIT dimer. Because spontaneous, SCF-independent dimerization of wild-type KIT (Figs 3a and b) caused neither autophosphorylation nor cytokine-independent growth in IC-2^WT^ cells, N508I mutation probably results in the conformational changes of KIT for activation. It also suggests that dimer formation does not always result in their activation, as reported in erythropoietin receptor and ErbB2/HER2 tyrosine kinases^37^,^38^,^39^. The glycosylated, mature form of KIT dimerizes in response to SCF binding, as observed with other receptor tyrosine kinases^40^,^41^,^42^. The phenomenon was also observed in a KIT variant with a mutation in the tyrosine kinase domain. Multiple bands with differing molecular weights were observed in western blots of IC-2^N508I^ cell lysates treated with BS_3_. This observation suggests that the N508I mutation induces aberrant dimer formation including the unglycosylated, immature form, which in turn may trigger abnormal downstream signaling and tumor promotion. The Tyr418 and Asn505 residues in the fifth Ig-like domain play a critical role in the dimerization of human KIT^34^. Because Asn508 in canine KIT corresponds to Asn505 in the human counterpart, a reasonable hypothesis is that the N508I mutation impairs the regulation of KIT dimerization, resulting in the constitutive KIT activation. Our results raise the possibility that mutations around either Tyr418 or Asn505 in human KIT can induce constitutive KIT dimerization and autophosphorylation. In agreement, most KIT mutants examined in this study exhibited higher circle values than wild-type KIT. In addition to the KIT receptor, equivalent mutations have been discovered in other receptor tyrosine kinases^22^,^23^,^43^,^44^,^45^. For example, extracellular domain mutations in the fibroblast-growth factor receptor 2 (FGFR2) have been implicated in congenital malformations, such as Crouzon or Apert syndrome^43^,^44^. Most FGFR2 mutations associated with these diseases are located in its third Ig-like domain, and Robertson *et al*.^45^ demonstrated that mutant FGFR2 variants dimerized in the absence of the natural ligand, as observed with the N508I mutant KIT in this study. Collectively, these data indicate that extracellular domain mutations, at least those in the Ig-like domains, can lead to the ligand-independent dimerization of receptors, resulting in the aberrant phosphorylation of various kinds of tyrosine kinase receptors.

Interestingly, treatment with KIT inhibitors increased the amount of activation-null KIT dimerization. Because neither STI571 nor AMN107 affected the *de novo* synthesis or internalization of KIT, inactive dimer formation may have resulted from physical interactions of these agents with KIT proteins. The degree of inactive dimer formation was not dependent on the kinase inhibitory potentials or chemical structures of those agents, but did depend on the concentration used. In addition, the increase in production of KIT dimers after STI571 or AMN107 treatment was comparable despite their distinct IC_50_ values on IC-2^N508I^ cells. The formation of inactive epidermal growth factor receptor (EGFR) dimers was observed when cells were treated with tyrosine kinase inhibitors that react with its active form^46^,^47^,^48^, suggesting these inhibitors induced an EGFR conformation similar to that of the activated state, without actually activating the kinase domain. Inactive KIT dimers formed by STI571 or AMN107 treatment may resemble inactive EGFR dimers. Though our results demonstrated the efficacy of tyrosine kinase inhibitors on N508I KIT, agent-dependent inactive dimer formations may modify outcomes in clinical cases by altering the duration of KIT turnover.

To the best of our knowledge, this is the first report demonstrating the tumorigenicity of an extracellular domain KIT mutation causing auto-dimerization in mast cells. These results aid in the understanding of the effects of KIT mutations, not only in mast cells, but also in other types of malignancies harboring mutations in tyrosine kinase-type receptors.

## Materials and methods

### MCTs and sequence analysis of c-*kit*

All dogs included in this study were previously referred for MCTs to the Animal Medical Center at Tokyo University of Agriculture and Technology. After appropriate surgical removal or fine needle aspiration of MCT specimens, total RNA from each sample was extracted by using Isogen (Nippon Gene, Toyama, Japan) and cDNA was synthesized with PrimeScript (Takara, Otsu, Japan). Polymerase chain reaction (PCR) was performed using a c-*kit*-specific forward primer (5′-GGA ATT CGC CAC CGC GAT GAG AGG CGC TCG CGG CGC CT-3′), a c-*kit*-specific reverse primer (5′-CTC TGC GGC CGC TCA CAC ATC TTC GTG TAC CAG CA-3′), and PrimeSTAR Max DNA Polymerase (Takara), according to the manufacturer’s instructions. The c-*kit* gene was sequenced using the BigDye Terminator v3.1 Cycle Sequencing Kit (Life Technologies, Gaithersburg, MD), forward primers corresponding to bases 243–263, 648–667, 1050–1069, 1484–1504, 1843–1862, 2205–2225, and 2639–2657, and a reverse primer corresponding to bases 386–406 (GenBank accession no. AF044249). Samples were analyzed using the ABI PRISM 3100-*Avant* Genetic Analyzer (Life Technologies). All experiments using clinical samples complied with the standards specified in the guidelines of the University Animal Care and Use Committee of the Tokyo University of Agriculture and Technology.

### Cell culture

The IC-2 mast cell line was a generous gift from Dr. Y. Kitamura (Osaka University, Osaka, Japan). IC-2 cells are phenotypically similar to mast cells, except that they lack KIT expression^14^. IC-2 cells were cultured in alpha-minimum essential medium (α-MEM; Life Technologies) supplemented with 10% fetal bovine serum (FBS), antibiotics, and 10 ng/mL recombinant murine IL-3 (R&D systems, Minneapolis, MN).

### Retroviral vector construction

Full-length wild-type c-*kit* cDNA was amplified using template cDNA from an HRMC mast cell line^49^, as described above. The PCR product was ligated into the pMXs-IRES-GFP plasmid (Cell Biolabs, San Diego, CA) using conventional methods and designated pMXs-KITWT-IRES-GFP. A plasmid encoding an Asn508Ile mutant (N508I) of c-*kit* was generated by site-directed mutagenesis and designated pMXs-KITN508I-IRES-GFP.

### Retroviral transfer

GP2-293 packaging cells (Clontech, Palo Alto, CA) were cotransfected with the pCMV-VSV-G plasmid (Cell Biolabs) and either the pMXs-IRES-GFP, pMXs-KITWT-IRES-GFP, or pMXs-KITN508I-IRES-GFP retroviral plasmid, using FuGENE 6 (Roche, Indianapolis, IN). The supernatant, containing replication-deficient virus particles, was used to infect IC-2 cells. The resulting IC-2 sublines expressing pMXs-IRES-GFP, pMXs-KITWT-IRES-GFP, or pMXs-KITN508I-IRES-GFP were termed IC-2^vector^, IC-2^WT^, and IC-2^N508I^ cells, respectively (Table 1). IC-2^N814V^ cells expressing the Asn814Val mutation (Table 1) were generated as previously described^50^. After establishment of these cell lines, all IC-2 sublines were maintained in α-MEM containing 10% FBS, antibiotics, and 10 ng/mL IL-3.

### Western blotting

After serum starvation for 12 h, cells were incubated for 4 h with 10 ng/mL SCF and/or 250 nM STI571 (Novartis Pharmaceuticals, Basel, Switzerland), and immunoblot analysis was conducted as described previously^51^. Primary antibodies including anti-phospho-KIT, anti-total/phospho-Akt, anti-total/phospho-S6 ribosomal protein, and β-actin antibodies purchased from Cell Signaling Technologies (Beverly, MA), and an anti-KIT antibody was purchased from Santa Cruz Biotechnology, Inc. (Santa Cruz, CA).

### Chemical crosslinking assay

IC-2 sublines were washed and resuspended in phosphate-buffered saline (PBS) containing 1 mg/mL bovine serum albumin. Cells were then incubated for 90 min at 4°C in the presence or absence of recombinant canine SCF and washed 3 times in PBS. In some experiments, cells were treated with STI571 or AMN107 (Selleck Chemicals, Houston, TX) at the indicated concentrations. Subsequently, cells were incubated for 30 min at 22°C in PBS containing 1 mM BS_3_ (Sigma-Aldrich Japan, Tokyo, Japan). Cross-linking reactions were terminated by washing the cells in ice-cold PBS, followed by incubation with 150 mM glycine-HCl (pH 7.5) for 5 min at 4°C. Western blot analysis was performed using lysates from these cells.

### WST assay

After serum deprivation for 12 h, cells were incubated with or without STI571 for 72 h. A WST assay was performed using the WST-8 Kit (Kishida Chemicals, Osaka, Japan) according to the manufacturer’s instructions. Fifty percent IC_50_ values were defined as the concentration of inhibitors at which 50% of cellular activation was attenuated compared to the control.

### Flow cytometry

To detect the expression of KIT receptors, cells were stained with an anti-KIT-APC antibody (clone 2B8, BioLegend, San Diego, CA). KIT-positive cells were detected with a MACSQuant flow cytometer (Miltenyi Biotec, Bergisch Gladbach, Germany). For cell cycle analysis, cells were harvested after a 24-h incubation with or without STI571 and fixed in 70% ice-cold ethanol, followed by treatment with RNase A and propidium iodide. Data were processed using the FlowJo FACS analysis software ver. 9.5.3 (Tree Star, Inc., Ashland, OR).

### Growth assessment of IC-2 sublines *in vivo*

All experiments with animals complied with both the standards specified in the guidelines of the University Animal Care and Use Committee of the Tokyo University of Agriculture and Technology and the guidelines for the use of laboratory animals provided by Science Council of Japan, as well as in accordance with Declaration of Helsinki, and were approved by the institutional committee. A total of 5 × 10^6^ IC-2 sublines were injected subcutaneously into the right and left flanks of 6-week-old female BALB/c-*nu/nu* mice (Charles River Japan, Yokohama, Japan). Tumors were measured with a caliper every 2 or 3 days. Tumor volumes (V) were calculated using the formula, V = ab^2^/2, where a and b are the length and width of tumor masses in mm, respectively. After 11 days of daily oral administration of 100 mg/kg STI571, mice were sacrificed and tumor tissues were used for immunohistochemical analysis.

### Immunohistochemistry

Histological analysis was conducted using IC-2^N508I^ tumor cells according to a previously described method^52^. An anti-phospho-KIT antibody (Abcam, Cambridge, UK) and an anti-Ki67 antibody (Abcam) were used as primary antibodies. Images were captured using a Nikon microscope (Nikon, Melville, NY).

### Structural modeling of the extracellular domain of KIT

Wild-type and mutant KIT conformations and KIT dimer stability were simulated using PDFAMS software (In-Silico Sciences Inc., Tokyo, Japan) by referencing the human c-*kit* gene, which shares approximately 80% homology with the canine c-*kit* gene^34^. The stability of the dimeric form was calculated by using circle values^35^. When 2 or more structures were compared by circle values, the structure with the highest value is more stable than the others^35^.

### Statistical analysis

A Mann-Whitney’s U test, Dunnett’s test, and a Student’s t-test were performed for statistical analysis of the data, and *p* values of > 0.05 were considered to be statistically significant.

## Author Contributions

Y.A., A.M., K.J., H.J., and S.I. performed experiments; Y.A. and K.O. analyzed data; Y.A., A.T., and H.M. designed the study and wrote the manuscript; and A.T. and H.M. supervised the study. All authors reviewed the manuscript.

## Supplementary Material

Supplementary Information Supplementary Information
