# Supplementary material for: A point mutation in the extracellular domain of KIT promotes tumorigenesis of mast cells via ligand-independent auto-dimerization
Source: Sci Rep. 2015 May 12;5:9775. doi: 10.1038/srep09775 (PMC4428273; doi:10.1038/srep09775)
Supplement: Supplementary Information [file srep09775-s1.pdf]

A point mutation in the extracellular domain of KIT promotes tumorigenesis of mast cells via ligand-independent auto-dimerization

Yosuke Amagai[1,2], Akira Matsuda[3], Kyungsook Jung[4,5], Kumiko Oida[1], Hyosun Jang[1], Saori Ishizaka[1], Hiroshi Matsuda[1,3*], and Akane Tanaka[1,4*]

[1]Cooperative Major in Advanced Health Science, Graduate School of Bio-Applications and System Engineering, Tokyo University of Agriculture and Technology, Tokyo, Japan

[2]Laboratory Animal Research Center, The Institute of Medical Science, The University of Tokyo, Tokyo, Japan

Laboratories of [3]Veterinary Molecular Pathology and Therapeutics and [4]Comparative Animal Medicine, Division of Animal Life Science, Institute of Agriculture, Tokyo University of Agriculture and Technology, Tokyo, Japan

[5]Eco-friendly Material Research Center, Korea Research Institute of Bioscience and Biotechnology, Jeonbuk, Korea

Supplementary information

KIT model	Circle values
wild-type	2.43
D419del	3.95
D419del_R420W_L421G	2.64
Y418R_D419del_R420W	2.35
Y418A_D419del	2.44
T417I_Y418-D419del	3.59
D419-R420del_L421F	3.06
T417V_Y418-D419del	2.97
T417R_Y418G_D419del	4.23
T417N_Y418-D419del	3.19
Y418-D419insFF_R420G	5.27
A502-Y503insYA	3.91

Table S1. Circle values of mutant human KITs. Based on the algorithms of PDFAMS software, the circle values of mutant human KIT reported by Kohl *et al.* [17] and Lux *et al.* [18] were calculated. To compare the stability of all mutant KITs, circle values were calculated taking into consideration 24 amino acid residues (414–429, 449, 489–491, and 501–504), which are located within 4.0 Å from residues 417–421 or 501–504.

Figure S1

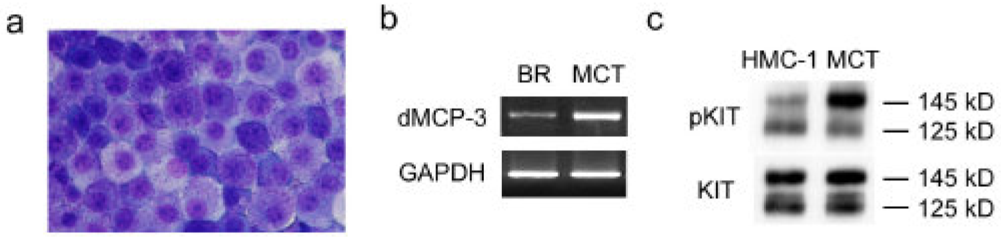

d

```
   1 ---------- ---------- --------at gagaggcact cgcggcgcct gggattttct
  61 ctgcgtcctg ctcctgctgc tgctgctcgg cgtccagaca ggctcttctc aaccatctgt
 121 gagtccaggg gaaccgtctc tcccatccat ccatccagca aaatcagagt taatagtcag
 181 tgtcggcgac gagcttaggc tgtcctgcac cgacccagga tttgtcaagt ggacttttga
 241 gaccctgggt caactgaatg agaacacaca caacgaatgg atcacagaga aggcagaggc
 301 tggccacacg ggcaattaca cgtgcaccaa cagagatggc ttgagcaggt ccatttatgt
 361 gtttgtcaga gatcctgcaa agcttttcct cgttgacctt cccttgtatg ggaaagaagg
 421 caatgatacg ctggtccgct gccctctgac ggacccagaa gtgaccaatt actccctcag
 481 ggggtgcgag gggaagcctc ttcccaagga cttgacgttc gtcgctgatc ccaaagctgg
 541 catcacgatc agaaacgtga agcgcgagta tcatcggctc tgcttgcact gctctgcgga
 601 ccagaagggc aggacggtgc tgtccaagaa attcaccctg aaagtgaggg cagccatcag
 661 agctgtacca gttgtgtcag tatccaaaac aagctctctc ctgaaggaag gggaagcctt
 721 ctctgtgatg tgctttataa aagatgtgtc tagtttcgtg gactcgatgt ggataaagga
 781 aaacagccag cagactaatg cacagacaca gagtaatagc tggcatcatg gtgacttcaa
 841 ttttgaacgt caggaaaagt tgattatcag ctcagcaaga gttaatgatt ctggagtgtt
 901 catgtgttac gccaataata cttttggatc agcaaatgtc acaacaacct tggaagtagt
 961 agataaagga ttcattaata tcttccccat gatgagtact acaatatttg taaatgatgg
1021 agagaatgtg gatctgattg ttgaatatga ggcatatccc aaaccggagc accagcagtg
1081 gatctatatg aacagaacct tcactgataa atgggaagat tatcccaagt ctgacaatga
1141 aagtaatatc agatatgtga gtgaacttca tctaaccaga ttaaaaggga acgaaggagg
1201 cacttacaca tttcaagtgt ccaattccga tgtcaattct tcggtgacat ttaatgttta
1261 tgtgaacaca aaaccagaaa tcctgactca tgaaagtctc acaaatggca tgctccagtg
1321 tgtggttgca ggattcccag agcccgcagt agattggtat ttctgtccag gagctgagca
1381 gagatgttct gtccctattg ggccaatgga tgtgcagatg caaaactcgt ctctgtcacc
1441 gtctggaaaa ctagtggttc agagttccat cgattatagt gccttcaagc acaatggcac
1501 agtcgagtgt agggcttaca acaatgtagg caggagttct gcctttttta Tctttgcatt
1561 taaagaacaa atccatcccc acaccctgtt cacacctttg ctgattggct ttgtgatcgc
1621 agctggaatg atgtgcatta tcgtgatgat tcttacctac aagtatctac agaaacccat
1681 gtatgaagta cagtggaagg ttgttgagga gatcaatgga aacaattatg tttacataga
1741 cccaacacag cttccttacg atcacaaatg ggagtttccc agaaacaggc tgagctttgg
1801 gaaaactttg ggtgctggtg ccttcgggaa agtggttgaa gccactgcat atggcctgat
1861 taagtcggat gcggccatga ctgttgccgt taagatgctc aaaccaagtg cccatttaac
1921 cgaacgagaa gccctaatgt ctgagctcaa agtcttgagt tacctcggta atcatatgaa
1981 tattgtgaat cttcttggag cgtgcaccgt tggagggccc accttggtca ttactgaata
2041 ttgttgctat ggtgatcttt tgaatttttt gcgaaggaaa cgtgattcat ttatttgctc
2101 aaagcaggaa gatcacggag aagtggcact ttataagaac cttctgcatt caaaggagtc
2161 ttcctgcagt gacagtacta atgaatacat ggacatgaaa cccggcgttt cttacgttgt
2221 gccaaccaag gcagacaaaa ggagatctgc gagaataggc tcatacatag aaagggatgt
2281 gactcctgcc atcatggaag atgatgagtt ggctctagat ctagaggact tgctgagctt
2341 ttcttaccag gtggccaagg gtatggcatt cctggcctcg aagaattgta ttcacagaga
2401 cttggctgct agaaatatcc tccttactca tggtcgaatc acaaagattt gtgattttgg
2461 tctagccaga gacatcaaga atgattctaa ttatgtggtc aaaggaaacg ctcggctacc
2521 tgtgaagtgg atggcccctg agagcatttt caactgtgtg tacacatttg aaagtgatgt
2581 ctggtcctat gggatttttc tgtgggagct cttctcttta ggaagcagcc cctaccctgg
2641 gatgccagtc gattcaaagt tctacaagat gatcaaggaa gggttccgga tgctcagccc
2701 tgagcatgca cctgctgaaa tgtatgacat catgaagacg tgctgggatg ctgatcccct
2761 gaaaaggccg acgttcaagc agatcgtgca gctaattgag aagcagattt cagatagcac
2821 caatcatatt tattccaacc tcgcgaactg cagccccaac ccagagcgcc ccgtggtgga
2881 ccattccgtg cggatcaatt ccgtgggcag cagcgcgtct tccacccagc ctctgctggt
2941 acacgaagat gtgtga
```

Figure S2

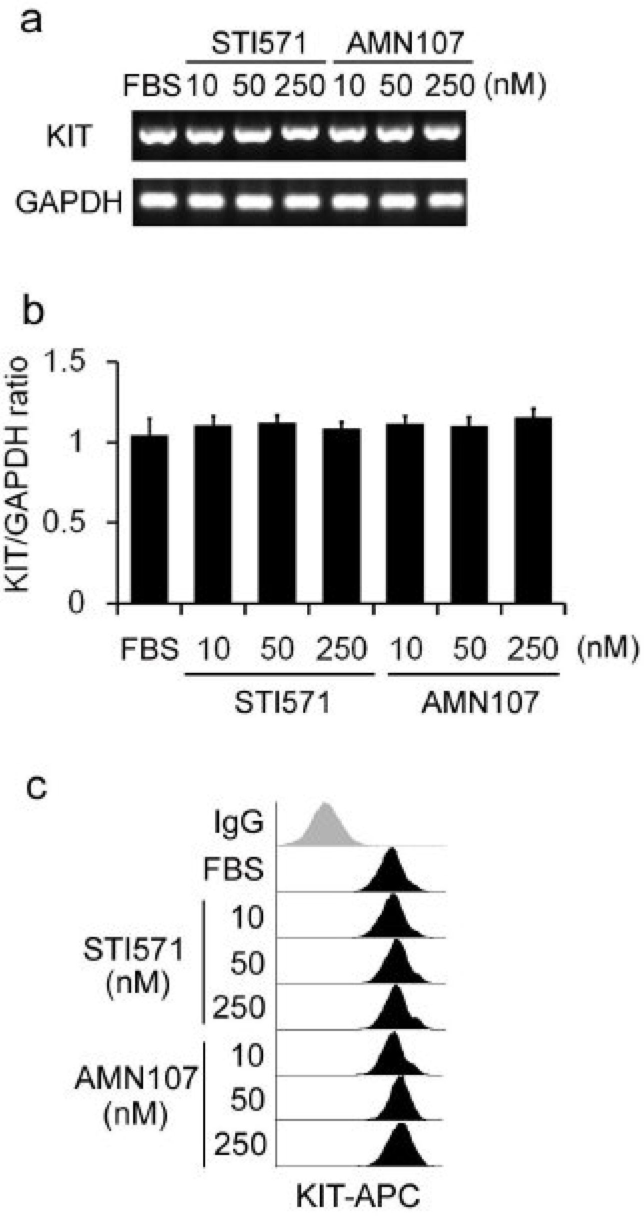
a
STI571
AMN107
FBS 10 50 250 10 50 250 (nM)
KIT
GAPDH
b
KIT/GAPDH ratio
1.5
1
0.5
0
FBS 10 50 250 10 50 250 (nM)
STI571
AMN107
c
IgG
FBS
STI571 (nM)
10
50
250
AMN107 (nM)
10
50
250
KIT-APC

Supporting figure legends

Figure S1. Characterization of a clinical MCT sample harboring the KIT Asn508Ile mutation. (**a**) Cytospin preparation of primary cultured tumor cells. A dog was diagnosed with multiple grade II MCTs occurring in both the mammary gland and perianal region. Primary tumor cells were cultured in AIM-V medium following collagenase digestion. The image shows a cytospin preparation of the tumor obtained from the mammary gland, which was stained with toluidine blue. Original magnification, ×200. (**b**) RT-PCR analysis of dMCP-3 expression in primary tumor cells. The data shown are representative of 2 independent experiments. The BR cell line is a tumor-derived canine mast cell line that was used as a positive control. (**c**) Western blot analysis of primary culture tumor cells. The phosphorylation status of KIT was examined using cell lysates of primary tumor cells grown in culture. HMC-1 cell lysates were used as a positive control. (**d**) The full length sequence of c-*kit* from a canine mast cell tumor with the 1551 A>T point mutation. Numbering corresponds to GenBank accession No. AF044249, and the capital "T" indicates the site of the 1551 A>T point mutation.

Figure S2. Effects of KIT inhibitors on *de novo* synthesis and internalization of KIT. (**a**) Expression of KIT mRNA in IC-2^{N508I} cells in the presence or absence of KIT inhibitors. RT-PCR analysis was performed using cells treated with the indicated concentrations of STI571 or AMN107 for 1 h. (**b**) The relative KIT/GAPDH expression ratios ± SD from 3 independent experiments are indicated. (**c**) Cell surface expression of the KIT receptor in IC-2^{N508I} cells in the presence or absence of KIT inhibitors. IC-2^{N508I} cells were treated with STI571 or AMN107 for 4 h at the indicated concentrations, stained with an anti-KIT-APC antibody, and analyzed by flow cytometry analysis. Data shown are representative of 3 independent experiments.
